# Dissociation between Simple and Complex Speech Motor Tasks within the Bilateral Motor Thalamus

**DOI:** 10.1523/ENEURO.0197-24.2025

**Published:** 2025-10-24

**Authors:** Karim Johari, Joel I. Berger, Andrea H. Rohl, Jeremy D. Greenlee

**Affiliations:** ^1^Human Neurophysiology & Neuromodulation Lab, Department of Communication Sciences and Disorders, Louisiana State University, Baton Rouge, Louisiana 70806; ^2^Human Brain Research Laboratory, Department of Neurosurgery, University of Iowa Hospitals and Clinics, Iowa City, Iowa 52242; ^3^Iowa Neuroscience Institute, University of Iowa, Iowa City, Iowa 52242

**Keywords:** deep brain stimulation, motor complexity, neural oscillations, speech production, vocalization

## Abstract

Although clinical and experimental evidence highlight the role of the thalamus in voluntary movement production, the involvement of the thalamus in complex motor tasks such as speech production remains to be elucidated. The present study examined neural activity within the bilateral thalamus in 13 participants (seven females) with essential tremor undergoing awake deep brain stimulation implantation surgery, using three speech tasks of varied complexity [vowel vocalization, a diadochokinetic task (DDK), and sentence repetition]. Low-frequency neural activity (delta/theta band) activity was significantly increased during sentence and DDK compared with vowel vocalization in the bilateral motor thalamus and, to a lesser extent, increased for sentence repetition compared with DDK. Moreover, there was prominent prespeech beta band activity, with a greater decrease in the power of beta activity for sentence compared with DDK and vowel vocalization. The greater low-frequency activity in more complex speech tasks may reflect the allocation of additional cognitive resources to monitor the execution of speech motor plans through cortico–thalamo–cortical pathways in a temporally precise manner. The greater decrease in the power of beta activity prior to the onset of sentence repetition may imply greater involvement of the bilateral thalamus in the planning of complex speech tasks. These findings provide new insights into the role of the bilateral thalamus in speech production and may have clinical implications for neurological disorders that affect speech production.

## Significance Statement

The cortical organization of speech production has been extensively investigated; however, the role of subcortical structures in speech remains unclear. The present study investigated the modulation of neural activity in the bilateral motor thalamus during three speech production tasks with varying complexity. Findings revealed differential modulation of theta and beta band activity in the bilateral motor thalamus for simple compared with complex speech tasks. The present study demonstrated that involvement of the bilateral thalamus depends on the complexity of the speech task and has translational implications.

## Introduction

Speech production is a complex motor task that depends on communication between cortical and subcortical regions in the human brain (Guenther, 2006; Tourville and Guenther, 2011). While the cortical organization of speech production has been broadly delineated in the literature (Hickok and Poeppel, 2007; Okada et al., 2018; Stark et al., 2019), the specific role of subcortical regions in speech production is not fully understood. Speech disorders are common in neurological conditions that affect subcortical regions such as Parkinson's disease (Ramig et al., 2007), which can impair different aspects of speech motor control including speech rate, fluency, loudness, and intelligibility (Goberman et al., 2010; Skodda, 2011).

The motor thalamus, one of the subcortical structures that is implicated in speech production (Guenther, 2006; Tourville and Guenther, 2011; Tankus et al., 2024b), receives inputs from brain regions involved in motor control including globus pallidus internus (GPI) and cerebellum (Sommer, 2003) and modulates activity in the premotor and motor cortex (Bosch-Bouju et al., 2013). Thalamic lesions cause speech deficits such as speech arrest and repetitions (Petrovici, 1980; Gorelick et al., 1984), possibly by compromised projections to those speech-related cortical areas. In addition, deep brain stimulation (DBS) of ventral intermediate nucleus of thalamus (VIM) in patients with essential tremor (ET), while improving motor symptoms (Nazzaro et al., 2013; Kondapavulur et al., 2022), can lead to stimulation-induced dysarthria (Lu et al., 2020; Kim et al., 2021). Therefore, an improved understanding of neural activity within the thalamus will elucidate the functional role of this region in speech production and may provide translational evidence to optimize stimulation parameters and minimize DBS-induced speech deficits following DBS of VIM.

Previous studies using intraoperative recording during awake DBS surgery report modulation of neural activity associated with speech production in subcortical structures (Lipski et al., 2017; Tankus and Fried, 2019; Tankus et al., 2021; Johari et al., 2023). Tankus et al. showed that left VIM single neurons modulated during vocalization, perception, and imaginary production of vowels (Tankus et al., 2024a). Using a machine learning algorithm, a high accuracy rate in identifying the patterns of single neurons was identified during vowel vocalization, perception, and imagination (Tankus et al., 2024b).

A recent study examined thalamic activity in beta, theta, and gamma frequency bands in response to reading written words and nonwords in patients with ET (Wang et al., 2022). Their findings suggested the role of the thalamus in lexical processing of words by demonstrating distinct patterns of modulations for reading words compared with nonwords. Although previous studies provide some insight into the role of the motor thalamus in speech production, we are not aware of any study which examines the role of thalamic activity in speech production tasks of varying motor complexity (i.e., complexity of the utterance). In this study, we addressed this knowledge gap by examining the thalamic activity associated with speech production of three tasks within the same participants: (1) vowel vocalization, (2) simple diadokinesis, and (3) sentence repetition. These tasks allowed us to probe whether neural oscillations within the bilateral thalamus would be modulated differently according to the complexity as a function of number of phonemes and number of articulator movements necessary for production of the utterance. As shown in a previous study (Wang et al., 2022), we expected that our tasks would elicit neural activity within low-frequency (delta/theta; 1–8 Hz) and beta (13–30 Hz) bands. Given the role of low-frequency activity within the thalamus and other cortical and subcortical structures in cognitive control and speech production (de Kloet et al., 2021; Domic-Siede et al., 2021; Singh et al., 2021), we hypothesized that these slower oscillations would also be modulated differently by task complexity, possibly reflecting more cognitive resources required for complex versus simple speech tasks. In addition, some evidence suggests that decreases in the power of premovement beta activity are modulated by movement complexity in other brain regions (Neuper et al., 2006; Tzagarakis et al., 2010; Johari and Behroozmand, 2020; Borra et al., 2023). Therefore, we hypothesized that our speech tasks would elicit premovement decreases in the power of beta activity, which would reflect motor planning and that this would modulate differently according to the complexity of the motor plan required. To test this hypothesis, we examined the role of bilateral thalamic activity during speech production by leveraging direct thalamic recordings during surgery in 13 participants with ET.

## Materials and Methods

### Participants

Thirteen individuals with ET (seven females; mean age, 59 years; Table 1) undergoing awake bilateral thalamic VIM DBS lead implantation surgery participated in the present study. Participants were native speakers of English. Formal neuropsychometry was not performed for these participants; all were high functioning and independently living. All participants provided written informed consent, and all study procedures were approved by the University of Iowa institutional review board and were in accordance with the Helsinki Declaration.

**Table 1.** Participant information

ID	Age	Sex	Handedness	FTM score^a^
1	72	Female	Right	NA
2	61	Male	Right	NA
3	60	Male	Right	117
4	64	Male	Right	38
5	42	Female	Right	NA
6	48	Male	Right	NA
7	25	Female	Left	44
8	72	Female	Right	NA
9	78	Female	Left	57
10	68	Female	Right	74
11	62	Male	Right	71
12	70	Male	Right	NA
13	67	Female	Right	80

a Fahn–Tolosa–Marin (FTM) tremor rating scale.

### Surgical procedure

During implantation surgery, bilateral VIM DBS leads were placed using standard, clinically necessary techniques. Briefly, framed stereotaxy was used for MRI-based targeting of VIM, and final lead positioning was refined and determined by clinical response to macrostimulation via the leads. Hand and upper extremity tremor reductions with transient contralateral hand paresthesias were noted in all participants to confirm that the deepest contact (Contact 1 here = Medtronic Contact 0) was positioned near the posteroinferior border of VIM. Postoperative CT scans were obtained in all participants and coregistered with preoperative MRIs to document lead locations and confirm appropriate thalamic placement. The MNI coordinates of the DBS leads were obtained for the right and left thalamus separately for each participant (Table 2).

**Table 2.** MNI coordinates of the distal contact of left and right DBS leads

Participant ID	Left			Right		
	*X*	*Y*	*Z*	*X*	*Y*	*Z*
546	−11	−16	3	14	−15	5
553	−13	−20	−6	14	−22	−7
541	−12	−27	−7	12	−23	−8
583	−12	−19	−7	14	−20	−10
599	−13	−18	−11	10	−16	−12
601	−14	−20	−7	13	−20	−6
604	−14	−18	−7	15	−18	−5
606	−13	−18	−7	15	−17	−11
607	−13	−19	0	12	−17	5
613	−13	−20	−2	14	−19	−1
616	−15	−18	10	11	−22	9
558	−11	−18	−5	16	−17	−9
582	−13	−27	7	13	−30	6

Note all electrodes are identical, with four contacts evenly spaced (1.5 mm).

### Speech and neural data

After satisfactory lead placement bilaterally and when all intraoperative intravenous sedation had worn off (e.g., dexmedetomidine), participants were instructed to perform interleaved vowel production (/α/), simple diadochokinesis (DDK; i.e., /tαtαtα/), and sentence repetition (“Buy Bobby a puppy,” /baɪ bɑbi ɑbpəpi/) tasks while neural data were recorded from the DBS leads. *Buy Bobby a puppy* was selected to limit memory-related cognitive load in the operating room while eliciting relatively complex tongue movements with multiple vowels including the diphthong /aɪ/ (Kleinow et al., 2001). These three speech tokens are frequently used in motor speech studies and vary in complexity according to the number of phonemes required (i.e., one unique phoneme for the sustained vowel, two for the DDK, six for “buy Bobby a puppy”) and number of articulator movements required (Kleinow et al., 2001; Skodda et al., 2011). Tasks were also selected in an effort to match approximate trial duration. Prior to surgery, participants had practice sessions to ensure they could perform the tasks. During this task introduction, participants were instructed to complete blocks of 10 trials with a pause or breath between each trial. A trial consisted of a single production of /α/ with a target duration of 1 s (note participants were instructed to sustain the vowel /α/ for ∼1 s), a production of the trisyllabic DDK /tαtαtα/, or a single production of “buy Bobby a puppy.” The tasks were modeled by the examiner, and the participant practiced with feedback until they were able to complete blocks of 10 trials independently. The task order was counterbalanced and verbally administered by a speech and language pathologist (SLP). In the operating room, the SLP verbally cued participants (e.g., “say tα-tα-tα 10 times”) to start and stop tasks. Because of the inherent unpredictability of patient response to the operating room environment, dynamic, responsive cueing was necessary to ensure sufficient usable trials for analysis. Cueing was exclusively verbal, following a structured cueing hierarchy aimed to minimize direction and modeling between trials. Shorter blocks and increased frequency of modeling were implemented as necessary due to alertness or timing concerns. Intertrial intervals varied [mean (SD), 1.5(0.6) s; min, 0.8 s; max, 3.1 s] according to participant performance and the level of independence with the task. Data collection time in the operating room was limited to a maximum of 15 min, and a range of 30–50 trials of each task were collected for each participant.

Neural data were simultaneously recorded from all contacts in the bilateral DBS leads. The first six participants were implanted with four-contact DBS leads in each hemisphere (3387, Medtronic). The subsequent seven participants received directional DBS leads with eight contacts in each hemisphere (B33015, SenSight, Medtronic). Both designs had 1.5 mm long contact lengths and 1.5 mm intercontact spacing along the lead shaft, while the middle two contact rows (Contacts 2, 3 here) were segmented into thirds in the SenSight lead. Since our analysis focused on local field potentials (LFP), which represent summed oscillatory activity from nearby neural populations, we averaged the three SenSight segments at the same depth into a single “contact” (i.e., 2A, 2B, 2C→“2”; 3A, 3B, 3C→“3”) for LFP analyses. This method therefore provided neural data from four contact depths in each hemisphere for each participant. Note we utilize Contact 1, 2, 3, and 4 nomenclature to correspond to Medtronic Contact 0, 1, 2, and 3 nomenclature, respectively. The neural data were referenced online to the left mastoid and recorded using a multichannel data acquisition system (TDT, Tucker-Davis Technologies) at a sampling rate of 2,000 Hz and high-pass filter of 0.3 Hz. An E6 Omnidirectional Earset microphone (Countryman Associates) fixed in position near the mouth was used to capture speech signals. The speech signal was digitally recorded using the TDT system at a sampling rate of 24,000 Hz simultaneously with neural data to provide a common timescale. The onset and offset of each trial were manually timestamped post hoc by the SLP in Praat (Boersma and Van Heuven, 2001).

### Data analysis

EEGLAB was used to preprocess neural data (Delorme and Makeig, 2004). First, each signal was downsampled to 500 Hz to speed processing. The signals were then submitted to the demodulated band transform toolbox (Kovach and Gander, 2016) to remove any line and/or transient noise contamination. The cleaned signals were then rereferenced to the average of four contacts in each hemisphere to remove common noise across electrode shafts (i.e., common average rereferencing). Finally, bandpass post hoc filtering between 1 and 50 Hz was done. Note that based on our hypotheses, we focused our analysis to theta and beta bands.

To evaluate perievent oscillatory activity (i.e., relative to speech onset), neural data were segmented from −2,000 before to 1,000 ms after the onset of speech tasks, and time–frequency analyses were performed on a trial-by-trial basis using a custom-made script in MATLAB (MathWorks). The epoch length of −2,000 to 1,000 ms was used to provide enough length to perform reliable spectral analyses for slow oscillations in delta/theta bands. However, for the statistical analysis, we focused on the time window of 500 ms before to 1,000 ms after the onset of the tasks. Due to the nature of the operating room environment, the duration of trials varied across participants and tasks (total number of trials across tasks, 1,581; mean duration, 1.3 s; median duration, 1.1 s). The median trial durations were 960 ms for vocalization, 1,270 ms for DDK, and 1,600 ms for sentence repetition. Based on these utterance durations, we limited our analysis from −500 ms to 1,000 ms after the onset of speech tasks to have comparable trial lengths across the tasks. All analyses were performed on speech onset-locked data.

**Figure 1.**
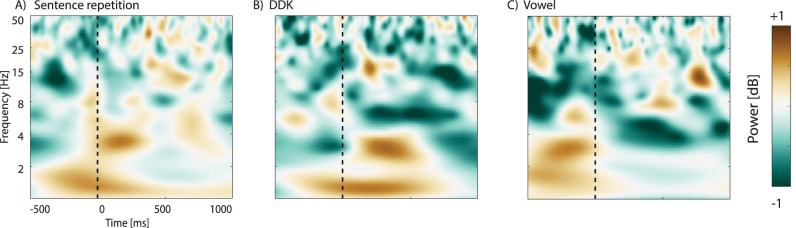
Unmasked time–frequency plots averaged for all participants within 1–50 Hz in response to (***A***) sentence repetition, (***B***) DDK, and (***C***) vowel vocalization from 500 ms before to 1,000 ms after the onset of the tasks.

Data were examined in the time–frequency domain by multiplying the fast Fourier transformed (FFT) power spectra of complex Morlet wavelets with the FFT single trial data (Cohen, 2014). The wavelet frequencies were logarithmically spaced, and the width of each frequency band gradually increased from 3 to 10 cycles as frequency increased from 1 to 50 Hz. The outcome of wavelet transformation was submitted to inverse FFT to obtain estimations of instantaneous power. For each frequency, the power values of each trial were normalized using the following formula: Power(dB) = 10 * log10 / (power_t_ − power_baseline_). The log transformation was done to ensure that data were normally distributed. In the formula, power_t_ represents the power at each time point, and power_baseline_ refers to the averaged power values from −2,000 to +1,000 ms. For each participant, the mean spectral response was calculated by averaging the power of each frequency across trials in each time point. Finally, the mean spectral response for each frequency was obtained across participants from −2,000 before to 1,000 ms after the onset of the tasks. The outcome of this spectral decomposition process was depicted as time frequency plots (Fig. 1).

### Statistical analysis

For within-task analyses, we compared the power of each frequency from 1 to 50 Hz using custom-written MATLAB scripts implementing EEGLAB's *bootstat* and *compute_pvals* functions (Delorme and Makeig, 2004). To examine individual electrode contacts within a single condition across participants, as well as between tasks across participants, data were shuffled 2,241 times within a baseline period (>−1,000 to ≤−500 ms) to create a surrogate distribution, in order to create values against which we could compare each for every time point and frequency. We then used *p* < 0.001 as the threshold for whether we could reject the null hypothesis that our observed data came from the surrogate distribution, based on the proportion of samples in the distribution that were smaller or larger than our observed values (i.e., two-tailed). For within-condition comparisons, these data were unsubtracted values observed for each participant. For between-condition comparisons (i.e., sentence-vowel, DDK-vowel, sentence-DDK), unthresholded data were first subtracted for each condition before performing the above statistical analysis.

### Interaction of laterality and contact locations

We did not have prior hypotheses as to whether the power of delta/theta and beta bands associated with speech complexity would interact differently with laterality and contact location. Therefore, a linear mixed-effects (LME) model was performed to examine the effect of laterality (left vs right thalamus) and contact locations (1–4) on the power of delta/theta and beta activity in prespeech and postspeech time windows. These analyses were performed on the mean power of delta/theta and beta oscillations compared with pairwise comparisons which were conducted at the resolution of 1 Hz. The choice of delta/theta and beta activity was based on the within-task and pairwise-comparison results (see above). For each frequency band, we included power as a dependent variable, task, laterality, and contacts as fixed factors and participants as a random factor, separately for prespeech (−500 to 0 ms) and during-speech (0–1,000 ms) time windows. For this analysis, Type 3 analysis of variance using Satterthwaite's method was reported for main effects and interactions. Follow-up analyses were performed using Bonferroni’s correction for multiple comparisons.

## Results

**Figure 2.**
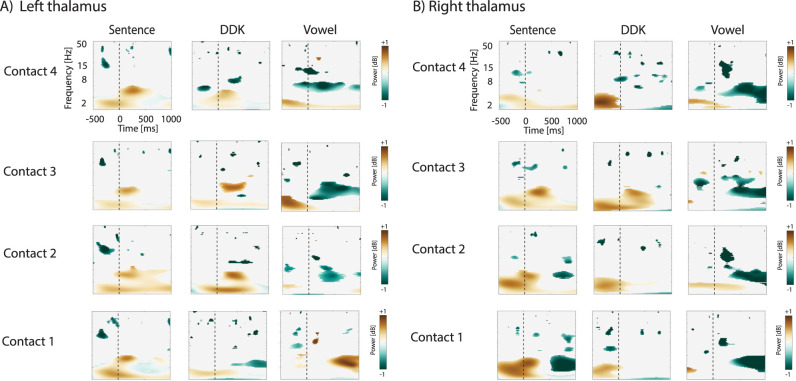
Averaged neural oscillations (1–50 Hz) within the left (***A***) and right (***B***) motor thalamus for 13 participants during sentence, DDK, and vowel vocalization tasks. The vertical line (−) denotes voice onset for each task. Each time–frequency plot is thresholded based on comparison to a surrogate distribution created from shuffling baseline values (*p* < 0.001; see Materials and Methods). Contact 1 is the deepest, Contact 4 is most superficial on the lead, and interelectrode distances are 1.5 mm.

Broadband recordings from bilateral thalamus in 13 participants revealed LFP activity during all three speech tasks, which were the largest amplitude in lower-frequency bands. Figure 2 shows statistically thresholded (*p* < 0.001) time–frequency plots for all participants in the left (Fig. 2*A*) and right (Fig. 2*B*) thalamus contacts across three tasks.

Across 13 participants, all tasks demonstrated an increase in delta/theta power in the bilateral thalamus. Additionally, a notable decrease in lower beta power (∼15 Hz) emerged prior to sentence repetition (Fig. 2).

### Pairwise comparisons

**Figure 3.**
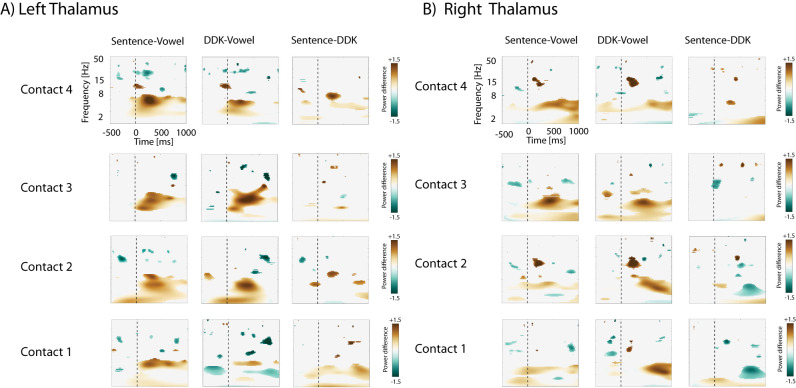
Pairwise comparisons of neural oscillations (1–50 Hz) associated with speech tasks averaged across participants, separately for the left (***A***) and right (***B***) thalamus. These comparisons are thresholded based on comparison to a surrogate distribution created from shuffling baseline values (*p* < 0.001; see Materials and Methods). The vertical line (−) represents voice onset of the speech tasks.

Pairwise comparisons indicated that sentences and DDK elicited greater increase in the power of delta/theta activity compared with vowel vocalization, particularly after speech onset. Figure 3 depicts the group results of pairwise comparisons between tasks from 1 to 50 Hz using an uncorrected threshold of *p* < 0.001, separately for the left (Fig. 3*A*) and right thalamus (Fig. 3*B*). Delta/theta power was also greater for sentence repetition compared with DDK after speech onset in the left thalamus contacts. Decreases in the power of lower beta activity (∼15 Hz) were greater for sentence repetition compared with vowel vocalization prior to speech onset. In contrast, beta power was not notably different for sentence versus DDK prior to speech onset.

### Effect of tasks, laterality, and contact locations

**Figure 4.**
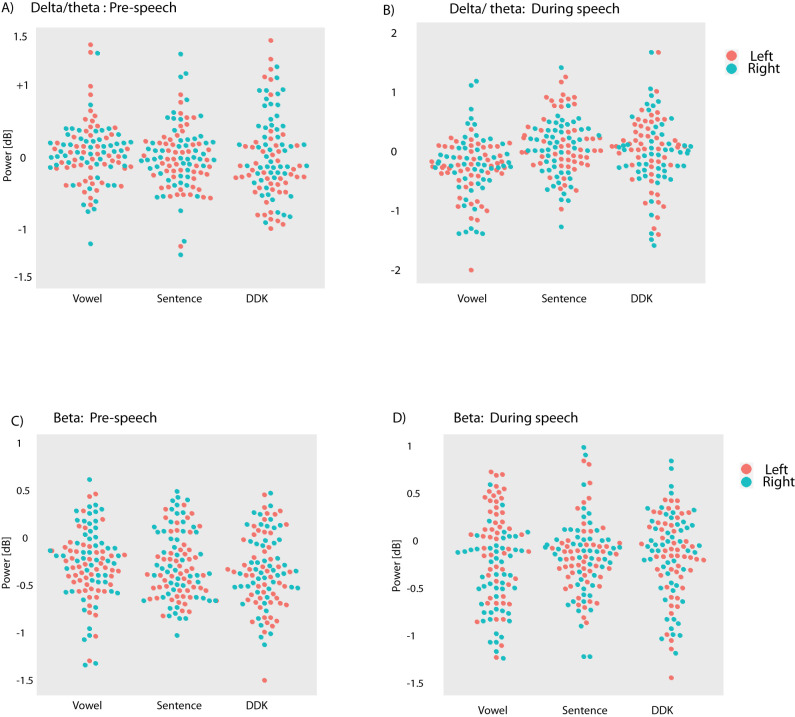
Bee swarm plots showing power of (***A***) prespeech theta, (***B***) during-speech theta, (***C***) prespeech beta, and (***D***) during-speech beta, separately for right (green circles) and left (red circles) thalamus contacts during speech tasks. Each panel shows data for all 13 participants. Note, there are 104 circles [2 (right and left VIM) × 4 (contacts) × 13 (participants)] for each task in all subpanels.

We performed an additional analysis to explore whether laterality and contact locations would interact with the power of delta/theta and beta activity before and after the onset of speech. LME results for prespeech theta activity did not yield any significant interactions between tasks, contact, and laterality (all *p* > 0.38). In addition, there were no significant effects of task (*F*_(2,276)_ = 0.42; *p* = 0.66; Fig. 4*A*) or contact (*F*_(6,276)_ = 0.54; *p* = 0.78). During-speech theta activity did not show a significant interaction between task, contacts, and laterality (all *p* > 0.24). However, there was a significant effect of task (*F*_(2,276)_ = 15.90; *p* < 0.0001), with an increase in the power of delta/theta after the onset of speech for sentence (*t*_(276)_ = 5.34; *p* < 0.0001; Fig. 4*B*) and DDK (*t*_(276)_ = −3.70; *p* = 0.0008; Fig. 4*B*) compared with vowel vocalization. There was no significant difference between DDK and sentence repetition in theta activity during speech production (*t*_(276)_ = 1.83; *p* = 0.21).

LME results for prespeech beta activity did not show interactions between task, laterality, and contact (*p* > 0.21). However, there was a significant effect of task (*F*_(2,276)_ = 3.22; *p* = 0.042), with a marginal difference between the power of the beta band for sentence versus DDK (*t*_(276)_ = 2.19; *p* = 0.09) and DDK versus vocalization (*t*_(276)_ = 2.20; *p* = 0.086). For beta power during speech production, there was a significant interaction between tasks and laterality (*F*_(2,276)_ = 8.20; *p* = 0.0003). Follow-up analysis indicated greater beta power on the right contacts for sentence (*t*_(276)_ = −3.42; *p* = 0.01) and DDK (*t*_(276)_ = −3.34; *p* = 0.014) compared with vowel vocalization (Fig. 4*D*). Furthermore, the left contacts showed greater beta activity compared with the right contacts during vowel vocalization (*t*_(276)_ = 4.83; *p* < 0.0001; Fig. 4*D*).

## Discussion

The present study investigated thalamic activity associated with three speech tasks of varying cognitive-speech demands. Specifically, sustained vowel vocalization, DDK, and sentence repetition tasks were used, as these are commonly used measures of speech performance in movement disorder patients and reflect increasing levels of motor speech complexity. We observed prominent delta/theta and beta modulations during planning and execution of all three speech tasks in the bilateral thalamus. However, there were significant differences across the three speech tasks in these oscillations, which highlight important aspects about the role of the motor thalamus in vocal motor production.

### Delta/theta activity

Low-frequency (1–8 Hz) activity has been reported during voluntary movements in humans (Tan et al., 2019; He et al., 2021). The severity of tremors in ET has been associated with the degree of the thalamic low-frequency (delta/theta) synchrony (Kane et al., 2009). This possibly reflects compromised long-range communication between the thalamus and motor and premotor cortices which could affect inhibitory processes during voluntary movement and subsequently lead to tremors (Malekmohammadi et al., 2015; Opri et al., 2019). In the present study, we demonstrated that three speech production tasks elicited low-frequency oscillations before and after the onset of the tasks. This is consistent with findings in Wang et al. (2022). They showed an increase in the power of low-frequency activity during reading aloud and suggested that this may reflect a long-range coupling communication between the thalamus and prefrontal cortex. We found that low-frequency activity was greater during sentence and DDK production compared with the vowel vocalization. This is novel evidence of bilateral thalamic activity differentially modulated by speech task complexity. The low-frequency activity increases may reflect an increase in the demand for long-range communication between the thalamus and motor and premotor cortex to plan and execute sequences of complex speech tasks. There is some evidence that speech planning and execution occurs as motor sequences (Bohland and Guenther, 2006; Bohland et al., 2010). According to this account, speech production requires the weighted activation of motor plans, which has been outlined in the GODIVA model of speech production (Guenther, 2006). GODIVA suggests that the supplementary motor area activates the motor plans for target words or syllables (Guenther, 2006; Tourville and Guenther, 2011; Chang and Guenther, 2020). According to this model, speech planning occurs in sequence of syllables. The thalamus and globus pallidus facilitate the cortical activations for the current motor plan (i.e., current syllable) by inhibiting the motor plans for the next syllable and then inhibiting the activated current motor plan once it is executed (Civier et al., 2013). Our findings that delta/theta oscillations in the thalamus were larger amplitude during sentence and syllable repetitions compared with simple vowel vocalization may reflect increase in the communication between the thalamus and the cortical regions in complex speech tasks that require activation and inhibition of several motor plans compared with simple vocalization tasks.

### Beta oscillations

Our tasks induced a decrease in the power of beta activity within the bilateral thalamus. Prespeech beta oscillations have been reported in cortical structures such as the premotor and motor cortex for both speech (Chrabaszcz et al., 2019) and limb movement (Kondylis et al., 2016; Lipski et al., 2017). Subcortical regions such as the thalamus and subthalamic nucleus have also demonstrated decreased beta activity during planning and execution of movements (Chrabaszcz et al., 2019; He et al., 2021; Basha et al., 2023), possibly reflecting disinhibition of selected motor plans in the motor and premotor cortex. Decreased thalamic beta activity occurring prior to our speech tasks is consistent with a recent study that showed similar pattern during words and nonwords reading (Wang et al., 2022). Beta activity was significantly decreased for sentences compared with vocalization and DDK tasks (Fig. 3). These findings together further highlight greater involvement of the thalamus in the facilitation of motor plans for complex speech tasks, such as sentence repetition, compared with less complex tasks within the same motor modality.

It is noteworthy to mention that our tasks did not show any effect of laterality and contact location, suggesting that involvement of the thalamus in our tasks was bilateral and not localized in certain subregions of the motor thalamus. Our DBS lead contact spacing provides coverage of VIM with the deepest two contacts, while Contacts 3 and 4 sit more proximally in ventralis oralis (Vo), anterior (Voa), and posterior (Vop) nuclei, which are also considered part of the “motor” thalamus. The Vo regions have overall similar connectivity as VIM, with projections to motor-related areas including the supplementary motor, premotor/prefrontal, and primary motor cortex (Hyam et al., 2012; Owen et al., 2022). However, Vo input is felt to be predominantly from GPI, while VIM input is predominantly cerebellar and VIM outputs are stronger to the primary motor cortex (Hyam et al., 2012). Our findings are in line with Wang et al. (2022) and suggest that speech production in tasks such as DDK and sentence repetition recruits all subregions of the motor thalamus (Wang et al., 2022).

The present study had several limitations. First, there is an increased prevalence of cognitive impairment in ET patients over the age of 65 (Janicki et al., 2013), and our participants were not formally tested with neuropsychometry. All participants were all high functioning and living independently without clinical concern for cognitive impairment. It is possible that some of our findings were affected by presurgical cognitive capacity. Moreover, we only used one syllable, one vowel, and one sentence to control the confounding effects of acoustic and lexicosemantic variability on thalamic activity associated with motor complexity across our speech, which limits generalizability. Tankus et al. reported differential firing patterns of thalamic neurons in response to five vowels (Tankus et al., 2024a) which implies articulator placement may impact firing patterns in the thalamus. The sentence used in our task is relatively simple and includes only bilabial consonants. Moreover, Wang et al. found that thalamic activity is sensitive to lexicosemantic processing of words (Wang et al., 2022). Future studies using additional, diverse speech tasks with varying semantic and articulatory content are required. For this study, task presentation relied on some degree of self-pacing for the participants, which increases the cognitive load of the task. We recognize that cognitive load may impact task performance and neural activity (Montero-Odasso et al., 2012). Task presentation and cueing was kept consistent between tasks within each participant, so these effects should be consistent across tasks. This is something that warrants further exploration, as our findings support that we may see greater thalamic involvement in response to cognitive load in much the same way we see differential responses to speech tasks depending on complexity, and it is unclear how much of these differences should be attributed to cognitive/semantic versus articulatory complexity.

Overall, the current study elucidates the specific role of thalamic oscillations in speech production and suggests that involvement of the thalamus depends on the task demands, by showing differential modulation of delta/theta and beta activity for complex versus simple speech tasks. We postulate that these thalamic oscillations may reflect dynamic interaction between cortical regions and the thalamus, in which the thalamus facilitates activated motor plans and inhibits them once they are produced. Future studies are required to fully explore this hypothesis and expand upon these findings, such as by recording simultaneous neural activity in the thalamus and cortical regions associated with speech production.

## Data Availability

The custom computational code used to generate the results in this manuscript is available upon request from the authors.

## Synthesis

Reviewing Editor: Alexander Soutschek, Ludwig-Maximilians-Universitat Munchen Decisions are customarily a result of the Reviewing Editor and the peer reviewers coming together and discussing their recommendations until a consensus is reached. When revisions are invited, a fact-based synthesis statement explaining their decision and outlining what is needed to prepare a revision will be listed below. The following reviewer(s) agreed to reveal their identity: NONE. Note: If this manuscript was transferred from JNeurosci and a decision was made to accept the manuscript without peer review, a brief statement to this effect will instead be what is listed below.

Reviewer #1

In the manuscript titled "Dissociation Between Simple and Complex Speech Motor Tasks Within Bilateral Motor Thalamus," the authors investigate how the complexity of speech tasks affects motor thalamus neural activities, specifically in the theta and beta frequency bands. The findings are a good complement to the current notion that thalamic neural activities are modulated based on task demands, specifically from the perspective of speech task complexity. I have the folllowing comments:

Major:

1. The authors discussed their findings on gamma activities in the Methods, Discussion, and Figure S1, which seem to diverge from established knowledge in this field. This could lead to confusion among readers and obscure the main findings of the present research. Consider removing these parts to ensure clarity and accuracy, and to prevent potential misinterpretation.

2. I would kindly ask the authors to reconsider using 'the first' in the significance statement and main text to emphasize the importance of the present study. While it might be true, the contribution of the present study to the knowledge in this field should not depend on this study being the first or not.

Minor:

1. In Introduction, "receiving inputs from Globus Pallidus Internus (GPI)" is not accurate, as the motor thalamus also receives inputs from the cerebellum.

2. In Table 1, I recommend placing the footnote as a standalone note below the table, rather than integrating it into the table.

3. In the legend of Figure 1 and throughout the main text, there seems to be a mixed use of two thalamic nuclei nomenclature systems (for example, Voa and Vop vs. ventral anterior nucleus and ventral lateral nucleus), which can be confusing. I suggest sticking with one, and provide clear full names before using abbreviations for clarity and readability.

4. In the Figure 2 legend, the results are described as based on all 13 participants, while the relevant main text above states that the Figure 2 results are for an exemplar subject. This is contradictory. Please clarify and make corresponding changes.

5. Figure 4 can be confusing due to mixed color coding and unclear labels. Consider showing left- and right-sided data separately in Figure 4A-C, similar to Figure 4D. In addition, suggest using 'Vowel' in place of 'Ah' and 'Syllable' in place of 'Ta' for better clarity.

Reviewer #2

The manuscript explores the modulation of neural activity in bilateral motor thalamus during speech production and found differential modulation of theta and beta band activity for simple compared to complex speech tasks.

Major concerns:

The manuscript presents the role of subcortical structures in general, and the thalamus in particular, during speech production as an unclear area, in contrast with the area of voluntary movements in this region. It completely ignores a large number of recent publications in both the thalamus and other subcortical areas, such as the subthalamic nucleus, that investigated speech production, perception and imagery at the single unit level, and provided details about the neuronal encoding schemes. Examples:

1. Tankus A, Lustig-Barzelay Y, Gurevitch G, Faust-Socher A, Strauss I. Neuronal Encoding of Speech Features in the Human Thalamus in Parkinson's Disease and Essential Tremor Patients. Neurosurgery. 2024;94(2):307-316.

2. Tankus A, Fried I. Degradation of Neuronal Encoding of Speech in the Subthalamic Nucleus in Parkinson's Disease. Neurosurgery. 2019;84(2):378.

3. Tankus A, Lustig Y, Fried I, Strauss I. Impaired Timing of Speech-Related Neurons in the Subthalamic Nucleus of Parkinson Disease Patients Suffering Speech Disorders. Neurosurgery. 2021;89(5):800-809.

Moreover, the neuronal representation has also been shown to allow the decoding of speech based on neural activity in both the thalamus and subthalamic nucleus:

4. Tankus A, Rosenberg N, Ben-Hamo O, Stern E, Strauss I. Machine Learning Decoding of Single Neurons in the Thalamus for Speech Brain-Machine Interfaces. J Neural Eng. 2024 9;21(3).

5. Tankus A, Solomon L, Aharony Y, Faust-Socher A, Strauss I. Machine learning algorithm for decoding multiple subthalamic spike trains for speech brain-machine interfaces. J Neural Eng. 2021;18(6):066021.

This literature is required in order to put the manuscript in context for the reader. In addition to literature survey, the manuscript should also discuss the differences between its findings and the ones already published.

Also, as the manuscript examined the production of 1 vowel ('Ah'), whereas Ref (1) above explored the 5 monophthongal vowels, please explain the differences and discuss how your new findings fit into the existing knowledge about vowel representation in the human thalamus.

Figure 3 and Pairwise comparisons:

The subtraction of responses in the frequency domain is presented along the time axis. However, there is a significant variability in task lengths. First, among patients (individual articulation durations vary). Second, between tasks. The "increased complexity" tasks also represent increased task duration: vowel is the shortest, syllable is longer, and a sentence is the longest. Sentence-vowel, for example, show differences in response that stretch along the whole 1 second after speech onset. As such, it may fully reflect the long sentence vs. a short 1 vowel articulation.

This may impose significant implications for the interpretation of the results. Please address directly the issue of the length of articulation, and take it into account in the discussion and interpretation of the results, as well as in the graphs indicating differences. If the difference is fully or partially because of the length of articulation, then the thalamus may not be claimed to be differentially involved in complex tasks in comparison with simple ones. The graphs must be controlled for task duration.

Less major issues:

Figure 1: Some contacts seem (from the figure) to lie outside the thalamus. Was this verified? Were these contacts ignored in analysis?

Methods - Please describe the paradigm in detail. What were the vowel and syllable used? Please provide the IPA transcription. What were the instructions to the patients? What were the verbal cues for onset? Verbal cues are usually longer than auditory ones (such as "beeps" used in Ref. 1 above). How was timing measured? A patient may react when starting to pronounce the instruction word, especially when repeating an instruction, whereas another patient may wait until the instruction was completely uttered. Was this monitored? This may introduce significant variation in measuring reaction times.

While time limitations in the OR are understandable, still the task seems to have only 1 sentence that patients repeated: "Buy bubby a puppy". Words in this sentence rhyme. Phonemes repeat. It has a very restricted set of phonemes. The word "bubby" shows affection. There are many specific variables to which the thalamus may respond. It is hard to determine, from the results, that the thalamus prefers complex speech tasks over simple ones. It may respond to an affection-related variable, which is definitely not a "complex speech task". Such variables should be controlled in the task (e.g., several types of complex sentences), or conclusions and title be rephrased.

Discussion, p. 12: "...three speech tasks of varying cognitive-speech demands" - As participants in the study are ET patients, please refer to their cognitive status. If their cognitive status is not fully preserved, can results be affected by their disease, rather than reflect the normal speech representation?

Minor concerns:

"statistically thresholding by shuffling 1000 times against a baseline period" (p. 7) - Please explain: what is thresholding by shuffling?

Figure 2: "Neural oscillations (1 to 50 Hz) ... for all 13 participants during ..." - The figure seem to depict only 1 graph for each contact / task / hemisphere combination, not 13. Is this the average? median? else? Please explain. What does "thresholded using permutation testing (p < 0.001)" mean? Please explain how the null distribution and threshold(s) is (are) computed.

Figure 4: What does the x-axis represent within a task? That is, for example, what makes one red point be plotted to the right of another red point? Please explain.

## Author Response

Author response to reviews of eN-TNWR-0197-24X, "Dissociation Between Simple and Complex Speech Motor Tasks Within Bilateral Motor Thalamus" All reviewer comments are itemized below, with our response to each in purple. Changes in the text are highlighted in yellow.

Computational Neuroscience Model Code Accessibility Comments for Author:

Please encode a code and data availability statement. This is now included.

REVIEWER #1 In the manuscript titled "Dissociation Between Simple and Complex Speech Motor Tasks Within Bilateral Motor Thalamus," the authors investigate how the complexity of speech tasks affects motor thalamus neural activities, specifically in the theta and beta frequency bands. The findings are a good complement to the current notion that thalamic neural activities are modulated based on task demands, specifically from the perspective of speech task complexity. I have the following comments:

Response: Thank you for your constructive feedback.

Major:

1. The authors discussed their findings on gamma activities in the Methods, Discussion, and Figure S1, which seem to diverge from established knowledge in this field. This could lead to confusion among readers and obscure the main findings of the present research. Consider removing these parts to ensure clarity and accuracy, and to prevent potential misinterpretation.

Response: We have now revised new figure 1 showing results up to 50 Hz, and revised discussion of gamma in the methods and discussion.

2. I would kindly ask the authors to reconsider using 'the first' in the significance statement and main text to emphasize the importance of the present study. While it might be true, the contribution of the present study to the knowledge in this field should not depend on this study being the first or not.

Response: The text has been revised as suggested by the reviewer.

Minor:

1. In Introduction, "receiving inputs from Globus Pallidus Internus (GPI)" is not accurate, as the motor thalamus also receives inputs from the cerebellum.

Response: The text has been revised to clarify based on the reviewer's comment.

2. In Table 1, I recommend placing the footnote as a standalone note below the table, rather than integrating it into the table.

Response: The footnote is placed below table 1.

3. In the legend of Figure 1 and throughout the main text, there seems to be a mixed use of two thalamic nuclei nomenclature systems (for example, Voa and Vop vs. ventral anterior nucleus and ventral lateral nucleus), which can be confusing. I suggest sticking with one and provide clear full names before using abbreviations for clarity and readability.

Response: To avoid confusion relating to nomenclatures and imprecision inherent in co-registration of individual patient anatomy into a combined series of MNI space, we have removed this figure.

4. In Figure 2 legend, the results are described as based on all 13 participants, while the relevant main text above states that the Figure 2 results are for an exemplar subject. This is contradictory. Please clarify and make corresponding changes.

Response: All plots show averaged data for all 13 participants; we have corrected the text and captions.

5. Figure 4 can be confusing due to mixed color coding and unclear labels. Consider showing left- and right-sided data separately in Figure 4A-C, similar to Figure 4D. In addition, suggest using 'Vowel' in place of 'Ah' and 'Syllable' in place of 'Ta' for better clarity.

Response: We have revised and improved the figures based on this helpful suggestion.

REVIEWER #2 The manuscript explores the modulation of neural activity in bilateral motor thalamus during speech production and found differential modulation of theta and beta band activity for simple compared to complex speech tasks.

Response: We thank the reviewer for the constructive comments.

Major concerns:

The manuscript presents the role of subcortical structures in general, and the thalamus in particular, during speech production as an unclear area, in contrast with the area of voluntary movements in this region. It completely ignores a large number of recent publications in both the thalamus and other subcortical areas, such as the subthalamic nucleus, that investigated speech production, perception and imagery at the single unit level, and provided details about the neuronal encoding schemes. Examples:

1. Tankus A, Lustig-Barzelay Y, Gurevitch G, Faust-Socher A, Strauss I. Neuronal Encoding of Speech Features in the Human Thalamus in Parkinson's Disease and Essential Tremor Patients. Neurosurgery. 2024;94(2):307-316.

2. Tankus A, Fried I. Degradation of Neuronal Encoding of Speech in the Subthalamic Nucleus in Parkinson's Disease. Neurosurgery. 2019;84(2):378.

3. Tankus A, Lustig Y, Fried I, Strauss I. Impaired Timing of Speech-Related Neurons in the Subthalamic Nucleus of Parkinson Disease Patients Suffering Speech Disorders. Neurosurgery. 2021;89(5):800-809.

Moreover, the neuronal representation has also been shown to allow the decoding of speech based on neural activity in both the thalamus and subthalamic nucleus:

4. Tankus A, Rosenberg N, Ben-Hamo O, Stern E, Strauss I. Machine Learning Decoding of Single Neurons in the Thalamus for Speech Brain-Machine Interfaces. J Neural Eng. 2024 9;21(3).

5. Tankus A, Solomon L, Aharony Y, Faust-Socher A, Strauss I. Machine learning algorithm for decoding multiple subthalamic spike trains for speech brain-machine interfaces. J Neural Eng. 2021;18(6):066021.

This literature is required in order to put the manuscript in context for the reader. In addition to literature survey, the manuscript should also discuss the differences between its findings and the ones already published.

Also, as the manuscript examined the production of 1 vowel ('Ah'), whereas Ref (1) above explored the 5 monophthongal vowels, please explain the differences and discuss how your new findings fit into the existing knowledge about vowel representation in the human thalamus Response: We have now cited and discussed the suggested references in the introduction and discussion.

Figure 3 and Pairwise comparisons:

The subtraction of responses in the frequency domain is presented along the time axis. However, there is a significant variability in task lengths. First, among patients (individual articulation durations vary). Second, between tasks. The "increased complexity" tasks also represent increased task duration: vowel is the shortest, syllable is longer, and a sentence is the longest. Sentence-vowel, for example, show differences in response that stretch along the whole 1 second after speech onset. As such, it may fully reflect the long sentence vs. a short 1 vowel articulation.

This may impose significant implications for the interpretation of the results. Please address directly the issue of the length of articulation and take it into account in the discussion and interpretation of the results, as well as in the graphs indicating differences. If the difference is fully or partially because of the length of articulation, then the thalamus may not be claimed to be differentially involved in complex tasks in comparison with simple ones. The graphs must be controlled for task duration.

Response: It is a valid point that there are differences in the utterance durations of single vowels vs sentences, and we were therefore missing information from the initial submission that was important in describing why this did not confound our interpretation of the results. We addressed the length discrepancy in both the task design and our analysis - participants were asked to repeat the syllables three times and hold their vocalization for about 1-2 seconds, while they were asked to repeat the sentence once. Across all trials and subjects, we had a median duration of 960 ms for vowels, 1270 ms for syllable and 1600 ms for syllable repetition. Therefore, we limited the analysis and plotting to up to the first 1000 ms to control for any length discrepancies between three tasks. We have now explained these details in the methods section.

Less major issues:

Figure 1: Some contacts seem (from the figure) to lie outside the thalamus. Was this verified? Were these contacts ignored in analysis? Response: We used intraoperative macrostimulation through the DBS leads to refine MRI guided targeting and position leads within VIM (see Methods). Post-op imaging was obtained in all participants to verify leads were in thalamus for each participant. To avoid confusion and anatomic imprecision of co-registration of individual patient anatomy into MNI space we have removed the anatomical figure.

Methods - Please describe the paradigm in detail. What were the vowel and syllable used? Please provide the IPA transcription. What were the instructions to the patients? What were the verbal cues for onset? Verbal cues are usually longer than auditory ones (such as "beeps" used in Ref. 1 above). How was timing measured? A patient may react when starting to pronounce the instruction word, especially when repeating an instruction, whereas another patient may wait until the instruction was completely uttered. Was this monitored? This may introduce significant variation in measuring reaction times.

Response: The SLP guided the patients to start or stop tasks. The speech signal was synchronized with the neural data and the SLP timestamped the onset and offset of subjects' responses in each trial. Intertrial intervals were calculated based on the offset of the last instance of speech, whether SLP or patient produced. We have now added these details to the method section.

While time limitations in the OR are understandable, still the task seems to have only 1 sentence that patients repeated: "Buy bubby a puppy". Words in this sentence rhyme. Phonemes repeat. It has a very restricted set of phonemes. The word "bubby" shows affection. There are many specific variables to which the thalamus may respond. It is hard to determine, from the results, that the thalamus prefers complex speech tasks over simple ones. It may respond to an affection-related variable, which is definitely not a "complex speech task". Such variables should be controlled in the task (e.g., several types of complex sentences), or conclusions and title be rephrased.

Response: We utilized the sentence "Buy Bobby a Puppy". This sentence has been used in previous research on motor speech in movement disorders and elicits relatively complex tongue movements and pronounced jaw movement in the context of a short, semantically simple utterance (Kleinow, Smith, &Ramig, 2001; Maner, Smith, &Grayson, 2000). The sentence does not contain any affection (we used 'Bobby' not 'bubby'). We agree with the reviewer that there might be variables that may interact with our main results. The main reason we did not include several vowels or syllables was to minimize the acoustic variability and make all trials as similar as possible. We also avoided using more than one sentence to control the confounding effects of variable lexical and semantic processing on neural activity since thalamus has been implicated in semantic and lexical processing. However, we acknowledge our choice of utterances as a limitation of this study in the revised manuscript.

Discussion, p. 12: "...three speech tasks of varying cognitive-speech demands" - As participants in the study are ET patients, please refer to their cognitive status. If their cognitive status is not fully preserved, can results be affected by their disease, rather than reflect the normal speech representation? Response: Thanks for raising this point. Although all of our patients were living independently and high functioning, not all of our patients had formal pre-surgical neuropsychological testing. We have now discussed this in the limitations of the study.

Minor concerns: "statistically thresholding by shuffling 1000 times against a baseline period" (p. 7) - Please explain: what is thresholding by shuffling? Response: Thank you for highlighting this. We have revised the text to clarify, as this was not correct in the way we had originally described it, which is true for the next comment as well. Baseline data (>-1000 ms to <=-500 ms) alone were in fact shuffled 2241 times along the time dimension, for each frequency and each participant, due to (1) the way the bootstat function determines the number of shuffles [i.e. if 2/the alpha value of 0.001 is greater than the number of shuffles, it overrides the desired input of 1000 shuffles and calculates the number of shuffles based on 2/0.001, equaling 2000] and (2) the way that the bootstat function indexes to create the surrogate array (it shuffles the times in chunks, meaning that it shuffles the 249 baseline timepoints (based on a 500 Hz sampling rate) nine times to meet the desired number of shuffles [i.e. 249 * 9 = 2241]. This created a surrogate data distribution of power changes during a baseline period against which we could compare all our observed values, to determine whether there were any significant differences over time for each frequency band (as in the case of the condition comparison, i.e. sentence vs vowel, syllable vs vowel, sentence vs syllable; Figure 3) or whether there was significant modulation over time for each frequency band (as in the case of the single condition; Figure 2). We used p < 0.001 as the threshold for whether we could reject the null hypothesis that our observed data come from the surrogate distribution, based on the proportion of samples in the distribution that were smaller or larger than our observed values (i.e. two-tailed).

Figure 2: "Neural oscillations (1 to 50 Hz) ... for all 13 participants during ..." - The figure seem to depict only 1 graph for each contact / task / hemisphere combination, not 13. Is this the average? median? else? Please explain. What does "thresholded using permutation testing (p < 0.001)" mean? Please explain how the null distribution and threshold(s) is (are) computed.

Response: As above, we appreciate the reviewer highlighting the lack of clarity here. Please see previous comment for correction of the permutation testing text. We have now revised the text accordingly. The figures are grand average of neural activity across the subjects.

Figure 4: What does the x-axis represent within a task? That is, for example, what makes one red point plot to the right of another red point? Please explain.

Response: We have now revised the figure based on the reviewer 1 comments. We have now added the tasks on the x- axis. Figure 4 shows the distribution of extracted power for both beta and theta before and during speech tasks. We have used Bee swarm plots to display the extracted power, which allows us to show the pattern of data and minimize the overlap between data points by placing data points next to each other.
